# Central Auditory Functions of Alzheimer’s Disease and Its Preclinical Stages: A Systematic Review and Meta-Analysis

**DOI:** 10.3390/cells11061007

**Published:** 2022-03-16

**Authors:** Hadeel Y. Tarawneh, Holly K. Menegola, Andrew Peou, Hanadi Tarawneh, Dona M. P. Jayakody

**Affiliations:** 1 Ear Science Institute Australia, Subiaco, WA 6008, Australia; hadeel.tarawneh@research.uwa.edu.au (H.Y.T.); holly.menegola@earscience.org.au (H.K.M.); andrew.peou@earscience.org.au (A.P.); hanadi.tarawneh@hotmail.com (H.T.); 2 Ear Sciences Centre, School of Surgery, University of Western Australia, Crawley, WA 6009, Australia; 3 School of Human Sciences, The University of Western Australia, Crawley, WA 6009, Australia; 4 WA Centre for Health and Aging, University of Western Australia, Crawley, WA 6009, Australia

**Keywords:** dementia, central-auditory processing, hearing loss

## Abstract

In 2020, 55 million people worldwide were living with dementia, and this number is projected to reach 139 million in 2050. However, approximately 75% of people living with dementia have not received a formal diagnosis. Hence, they do not have access to treatment and care. Without effective treatment in the foreseeable future, it is essential to focus on modifiable risk factors and early intervention. Central auditory processing is impaired in people diagnosed with Alzheimer’s disease (AD) and its preclinical stages and may manifest many years before clinical diagnosis. This study systematically reviewed central auditory processing function in AD and its preclinical stages using behavioural central auditory processing tests. Eleven studies met the full inclusion criteria, and seven were included in the meta-analyses. The results revealed that those with mild cognitive impairment perform significantly worse than healthy controls within channel adaptive tests of temporal response (ATTR), time-compressed speech test (TCS), Dichotic Digits Test (DDT), Dichotic Sentence Identification (DSI), Speech in Noise (SPIN), and Synthetic Sentence Identification-Ipsilateral Competing Message (SSI-ICM) central auditory processing tests. In addition, this analysis indicates that participants with AD performed significantly worse than healthy controls in DDT, DSI, and SSI-ICM tasks. Clinical implications are discussed in detail.

## 1. Introduction

Globally, 55 million people were estimated to be living with dementia in 2020 [1]. With the ever-growing aging population, this number is projected to increase to 78 million in 2030 and 139 million in 2050 [2]. Dementia is a clinical syndrome caused by neurodegeneration, Alzheimer’s disease, vascular dementia, Lewy body, and frontotemporal dementia and characterised by a progressive decline in cognitive and daily living functions [3]. Alzheimer’s disease (AD) accounts for 60–80% of dementia cases [1]. AD is characterised by the deterioration or decline in episodic memory function, attention skills, visual-spatial orientation, judgement, abstract thinking, and language skills [4]. The physical and neurological symptoms of AD are caused by neuropathological changes to the brain in the form of neurofibrillary tangles and amyloid plaques [5]. The rate of decline for someone with AD can vary significantly. However, neuropathological changes can begin to manifest up to 20–30 years prior to clinical symptoms, which provides a unique opportunity for early intervention [6,7].

Approximately 40% of dementia diagnoses could be prevented or delayed by modifying early life (education), midlife (hypertension, obesity, hearing loss, traumatic brain injury, and alcohol misuse), and later life (smoking, depression, physical inactivity, social isolation, diabetes, and air pollution) risk factors [7]. Of the modifiable risk factors, midlife hearing loss contributes to a weighted population attributable fraction of 8.2%; however, current evidence suggests that as the severity of the peripheral hearing loss increases from mild to severe, the dementia risk also increases from double to five times [8].

There are two main components of the auditory system: the peripheral system and the central auditory system [9,10]. The peripheral hearing system consists of external, middle, and inner ears and cochlear nerve and is responsible for simple auditory tasks, such as detecting sound [10]. The acoustic information from the cochlea travels to the auditory cortex via the superior olivary complex, lateral lemniscus, inferior colliculus, and medial geniculate nucleus [9]. The auditory cortex is located on the superior temporal gyrus in the temporal lobe and contains a precise tonotopic map of the cochlea [9]. It has several defined regions: AI (primary including Brodmann area 41) and AII (secondary areas including Brodmann area 42, anterior, ventral, ventral-posterior, and posterior auditory fields) [11]. The A1 plays a crucial role in processing temporal information of complex signals such as speech and music [12], sound localisation [13], and source identification in auditory scene analysis [14]. The hippocampus detects novel acoustic signals and suppresses redundant auditory information [15]. Findings from animal studies reveal that fields anterior and ventral to A1 contain cells that respond to frequency and amplitude modulated tones and noise. Areas posterior to A1 have cells that respond to broader frequency tuning, longer tone response latencies, lower following rates of acoustic frequency, and amplitude modulations [16]. The posterior part of the superior temporal gyrus and the deeper planum temporale is known as the Wernicke’s area, which plays a vital role in phonological and lexical recognition [17]. A meta-analysis on neuroimaging studies revealed an activation likelihood of bilateral superior temporal cortices during an acoustic analysis of speech; left mid-posterior superior temporal sulcus for phonetic and phonological analysis and the left inferior parietal lobule in differentiating phoneme categories [18]. In summary, the central auditory pathway involves complex tasks such as auditory discrimination, temporal processing, and binaural processing, including sound localisation and lateralisation, as well as interpreting information in the presence of competing or degraded acoustic signals [19].

In current clinical practice, brain imaging techniques such as structural neuroimaging with magnetic resonance imaging (MRI) and molecular neuroimaging with positron emission tomography (PET) are used to support the diagnosis of AD [20,21]. Due to non-invasiveness and ease of accessibility, brain MRI is routinely used in clinical practice [22]. During the early stages of AD, atrophy in the brain is first observed in medial temporal lobe structures, including the entorhinal cortex (ERC) and hippocampus [23,24]. As the disease progresses, atrophy advances to the remainder of the medial temporal lobe [25,26]. Medial temporal lobe atrophy can differentiate AD from dementia with Lewy bodies [27] and predict those who will convert from MCI to AD [28]. Recent MRI studies have found that parietal lobe atrophy can also differentiate between AD and other dementias, including dementia with Lewy bodies and frontotemporal lobe degeneration [29]. As mentioned above, the neurodegenerative changes that affect both temporal and parietal lobes significantly impact the processing of complex acoustic signals such as speech. Hence, it is no surprise that one of the most common clinical features of central auditory processing (CAP) impairment is poor speech understanding in the presence of background noise or speech [30]. However, speech perception also depends on several cognitive functions (i.e., working memory, attention, executive functions) [31]. Hence, CAP impairment underlies the complex relationship between peripheral hearing sensitivity, auditory and speech perception, and cognitive abilities of older adults. Peripheral hearing loss is the second highest cause of disability globally, affecting 1.5 billion people, with 90% of cases due to age-related hearing loss [32]. Over 65% of adults above 60 years have some degree of peripheral hearing loss, with 58% experiencing moderate or higher-grade peripheral hearing loss [32]. Many studies have established an association between age-related peripheral hearing loss, cognitive impairment, and dementia [8,33,34,35]. In the absence of a severe peripheral hearing loss, age-related CAP impairment is associated with high incidences of cognitive decline and AD dementia [30,36,37]. Hence, age-related CAP impairment is considered a strong marker for increased risk of cognitive decline and AD [38,39]. AD pathology also commonly compromises the central auditory pathway, meaning that age-related CAP impairment may be a direct symptom of neurodegeneration [39,40,41]. Therefore, age-related CAP impairment could be a non-invasive early biomarker for cognitive decline and preclinical AD if a sensitive test battery was produced.

Given the higher prevalence of peripheral hearing loss in older adults, it is vital to establish the impact of peripheral hearing impairment in age-related CAP impairment. The central auditory pathway undergoes pathophysiological changes due to healthy ageing, i.e., a decline in temporal lobe volume [42], hippocampal volume [42], medial temporal lobe [43], prefrontal cortex [44], and brain volume [45]. Neuroimaging studies have reported a higher rate of decline in the entire brain volume and right temporal lobe volume [46], grey matter volume in superior and middle temporal gyri [47], and primary auditory cortex [48,49] due to age-related hearing loss. Hence, quantifying the impact of the severity of peripheral hearing loss on age-related CAP functions poses a challenge. Yet, the notion that age-related CAP impairment increases the risk of cognitive impairment in older adults remains unchanged.

The purpose of this paper was to systematically review currently available literature that examines the behavioural central auditory assessments of individuals aged 40–85 years to determine (i) challenges about selection and administration of behavioural central auditory processing assessments to those with impaired cognitive and/or daily living functions, (ii) modifications to the standard central hearing assessment procedures to accommodate impaired cognitive and/or daily living functions, and (iii) the factors that influence the interpretation of test results. Two research questions were investigated: (i) What are the most commonly utilised behavioural central auditory processing tests for older adults diagnosed with AD or in preclinical stages, and which are the most sensitive? (ii) What are the limitations of these measures, and how can they be improved? The primary objective is to identify a CAP assessment/test battery that is accurate and is a sensitive early detection tool for AD. This systematic review will contribute to understanding the strengths and limitations of behavioural central auditory function assessments in individuals with AD and those at risk of developing AD. It may also aid in identifying a method for early AD detection. At-risk individuals could be effectively screened and better referred for further neuropsychological assessments.

## 2. Methods

The methodological approach for this review is outlined in detail in the review protocol [50]. Methods were in accordance with Cochrane guidelines for systematic reviews [51]. All publications related to behavioural central auditory functions and AD dementia, mild cognitive impairment (MCI), and subjective cognitive decline (SCD) in adults published and available on searched databases before 15 February 2022 were considered. The search was performed on six major electronic databases; to maximise search strategy sensitivity, the following were used: MeSH terms in exploded mode (MEDLINE, PsychINFO and EMBASE), text searches or keywords (PubMed, CINAHL Plus and Scopus), with truncations, synonyms, and different spellings. Using the Comprehensive Meta-Analysis software version 3, a random-effects meta-analysis and analysis of study heterogeneity were conducted for this review. This systematic review followed the Preferred Reporting Items for Systematic Review and Meta-Analysis (PRISMA) statement [52] and is registered in PROSPERO (CRD42017078272).

## 3. Results

### 3.1. Search Results

**Figure 1:**
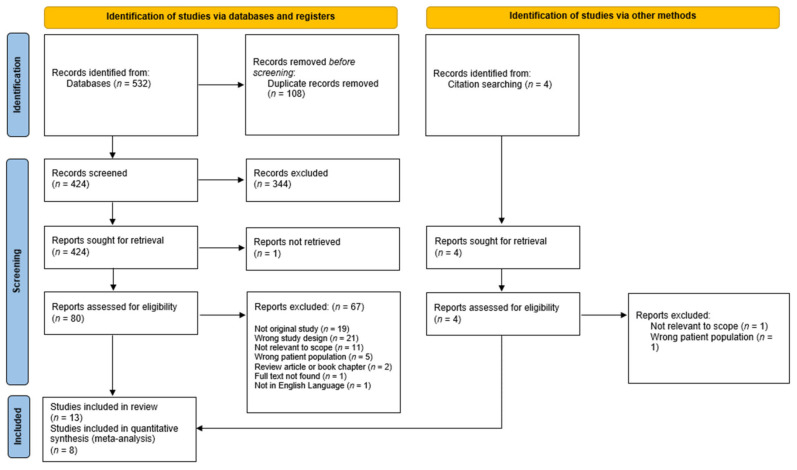
PRISMA flow chart of search results.

Figure 1 illustrates the selection of studies for the qualitative synthesis and the meta-analysis for this review. In total, 424 articles were found through the database and manual search following the removal of duplicates. Following title and abstract screening against the eligibility criteria, 344 articles were deemed irrelevant and excluded. Eighty articles were retrieved in full text to be examined; 67 were excluded for not meeting the full inclusion criteria. Thirteen studies met the full inclusion criteria and were included in the qualitative synthesis, and seven were included in the various meta-analyses.

### 3.2. Study Characteristics

Table 1 summarises the main study characteristics of the articles included in the systematic review. Among the studies included in the qualitative synthesis, four were from the USA, two from Egypt, one from the Netherlands, one from Sweden, one from Greece, one from Iran, one from Korea, one from Italy, and one from Australia. The pooled sample included 2561 participants with a total of 81 participants with SCD, 703 with incident MCI, 81 with AD dementia, and 1696 control participants. Studies used a variety of cognitive assessments and diagnostic criteria to diagnose cognitive impairment, these included cognitive ability screening instrument (CASI), clinical dementia rating (CDR), National Institute of Neurological and Communicative Diseases and Stroke–Alzheimer Disease and Related Disorders Association criteria (NINCDS-ADRDA), Diagnostic and Statistical Manual of Mental Disorders (DSM), Cambridge Cognitive Examination (CAMCOG), Petersen’s criteria, Mini-Mental State Examination (MMSE), Memory Assessment Clinics Questionnaire (MAC-Q), and the Montreal Cognitive Assessment (MoCA).

The studies included in this review evaluated 16 different behavioural central auditory assessments. Five studies used the synthetic sentence identification-ipsilateral competing message (SSI-ICM) test and the dichotic digits test (DDT) to evaluate central auditory function. Four studies used the dichotic sentence identification test (DSI), and three used the speech perception in noise test (SPIN). Adaptive tests of temporal response (ATTR), time-compressed speech test (TCS), auditory fusion test (AFT), auditory memory battery of Goldman–Fristoe–Woodcock (GFW), pitch pattern sequence (PPS), and the gap-in-noise (GIN) test were each evaluated in two studies to assess central auditory function. Synthetic sentence identification-competitive contralateral message (SSI-CCM), tone duration discrimination (TDD), speech in Babble (SinB), random gap detection test (RGDT), selective auditory attention test (SAAT), Quick Speech-in-Noise (QuickSIN), and duration pattern test (DPT) were each used in one study to assess central auditory function

### 3.3. Quantitative Analysis

Multiple central auditory processing tests were assessed on the basis of the pooled data of the studies included in the review. Meta-analyses were conducted on temporal auditory processing tests (ATF and ATTR), dichotic tests (DDT and DSI), and monaural low-redundancy speech tests (SSI-ICM, TCS, and SPIN). The results of the meta-analyses are reported as the standard difference in mean (SMD) with 95% confidence intervals (CI) as the synthesised measure of effect size. Heterogeneity was tested using Cochrane’s Q-value statistic, and the I-squared ($I^{2}$) statistic was performed to indicate heterogeneity as a percentage. For *I*^2^, values from 0% to 40% were considered low heterogeneity, 41% to 60% moderate, and >61% highly heterogeneous [53].

#### 3.3.1. Auditory Temporal Processing Tests

**Figure 2:**
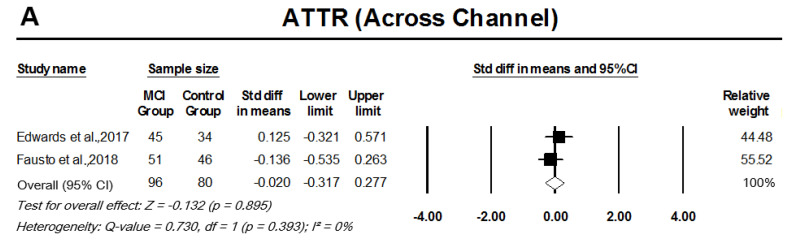

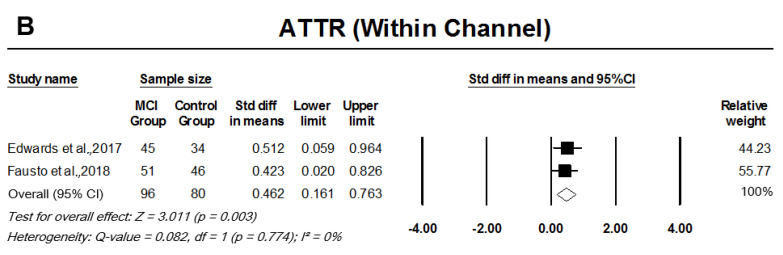
Forest plot of standard mean difference and overall (pooled) estimate of studies investigating adaptive tests of temporal resolution (ATTR) that compared a mild cognitive impairment (MCI) group to a control group. (**A**) Analysis of within-channel ATTR, (**B**) analysis of across-channel ATTR. Notes: Z = Z score; *I*^2^ = percentage of heterogeneity; Q-value = Cochrane’s Q; df = degrees of freedom. The horizontal lines represent the 95% confidence interval (CI) for each computed standard mean difference. Weights are from the random-effects analysis.

**Figure 3:**
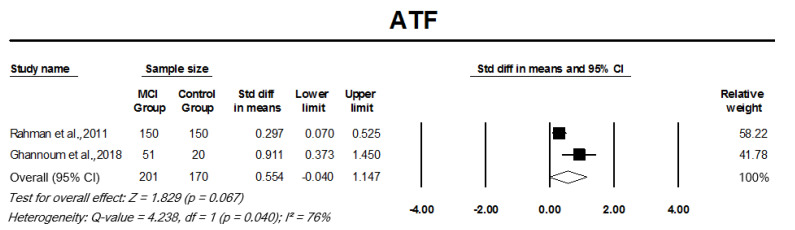
Forest plot of standard mean difference and overall (pooled) estimate of studies investigating auditory fusion test (ATF) that compared a mild cognitive impairment (MCI) group to a control group. Notes: Z = Z score; *I*^2^ = percentage of heterogeneity; Q-value = Cochrane’s Q; df = degrees of freedom. The horizontal lines represent the 95% confidence interval (CI) for each computed standard mean difference. Weights are from the random-effects analysis.

The meta-analysis of two cohort studies indicated a significant SMD between MCI participants and controls for within channel ATTR, pooled SMD, 0.46 (95% CI: 0.16–0.76, *p* = 0.003; Figure 2A). Across-channel ATTR, on the other hand, showed no significant difference between the results for MCI participants and controls, pooled SMD, −0.02 (95% CI: −0.32–0.28, *p* = 0.895; Figure 2B). There was no significant heterogeneity among the across-channel and within-channel ATTR studies ($I^{2}$ = 0%, *p* = 0.393 and $I^{2}$ = 0%, *p* = 0.774, respectively). Pooled analysis of ATF studies indicated that although there was moderate effect size, there was no significant difference between MCI participants and controls in mean ATF scores, pooled SMD, 0.55 (95% CI: −0.40–1.147, *p* = 0.067; Figure 3). The AFT studies had a significant heterogeneity, $I^{2}$ = 76%, *p* = 0.04.

#### 3.3.2. Dichotic Tests/Binaural Interaction Tests

**Figure 4:**
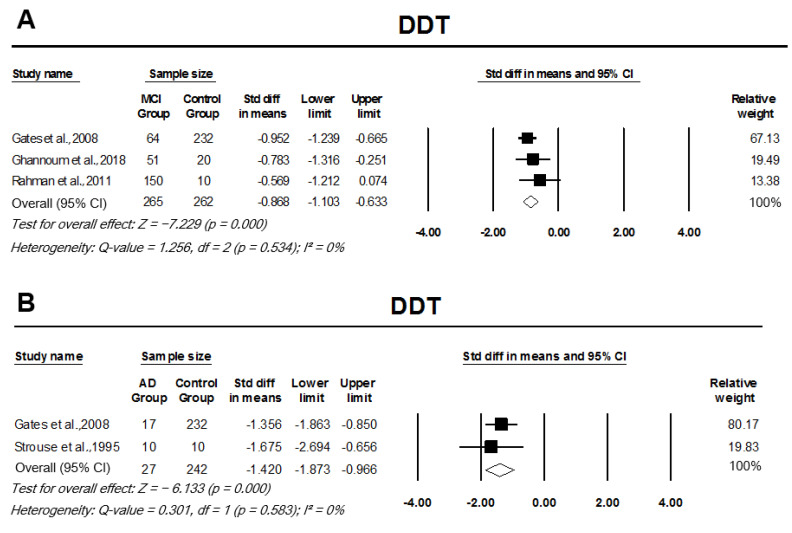
Forest plot of standard mean difference and overall (pooled) estimate of studies investigating dichotic digits test (DDT). (**A**) Analysis of DDT between the mild cognitive impairment (MCI) group and the control group, (**B**) analysis of DDT between the Alzheimer’s disease (AD) group and the control group. Notes: Z = Z score; *I*^2^ = percentage of heterogeneity; Q-value = Cochrane’s Q; df = degrees of freedom. The horizontal lines represent the 95% confidence interval (CI) for each computed standard mean difference. Weights are from the random-effects analysis.

**Figure 5:**
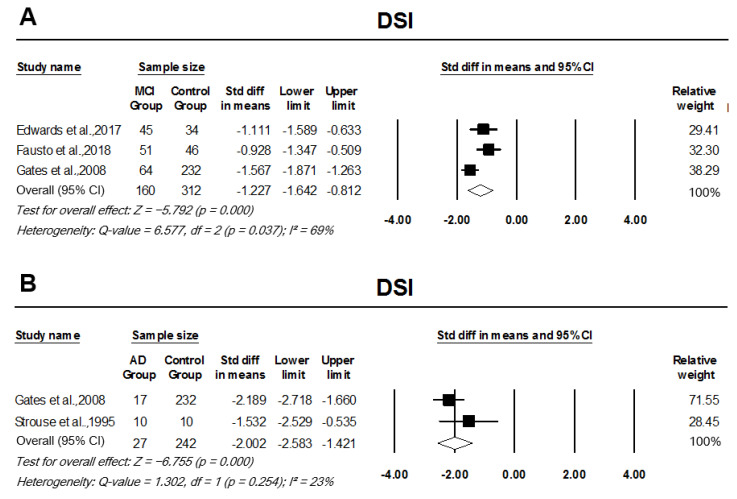
Forest plot of standard mean difference and overall (pooled) estimate of studies investigating dichotic sentence identification (DSI) test. (**A**) Analysis of DSI between the mild cognitive impairment (MCI) group and the control group; (**B**) analysis of DSI between the Alzheimer’s disease (AD) group and the control group. Notes: Z = Z score; *I*^2^ = percentage of heterogeneity; Q-value = Cochrane’s Q; df = degrees of freedom. The horizontal lines represent the 95% confidence interval (CI) for each computed standard mean difference. Weights are from the random-effects analysis.

In comparison to controls, participants with MCI performed seven deviations below the mean in DDT (z = −7.23), with a significantly large effect size, pooled SMD, −0.87 (95% CI: −1.10–−0.63, *p* = 0.000; Figure 4A). There was a significant SMD between AD participants and controls in the pooled analysis of DDT results, SMD −1.42 (95% CI: −1.83–−0.97, *p* = 0.000; Figure 4B). There was no heterogeneity across DDT studies comparing MCI participants or AD participants to controls, $I^{2}$ = 0%, *p* = 0.534 and $I^{2}$ = 0%, *p* = 0.583, respectively. Compared to controls, participants with MCI and AD scored significantly lower in DSI testing, with analysis indicating a large effect size, pooled SMD, −1.23 (95% CI: −1.64–−0.81, *p* = 0.000; Figure 5A) and −2 (95% CI: −2.58–−1.42, *p* = 0.000; Figure 5B), respectively. Studies looking at MCI participants’ DSI scores in comparison to controls had significant heterogeneity across the studies ($I^{2}$ = 69%, *p* = 0.037); however, between the AD group and controls, there was no significant heterogeneity ($I^{2}$ = 23%, *p* = 0.254).

#### 3.3.3. Monaural Low-Redundancy Speech Tests

**Figure 6:**
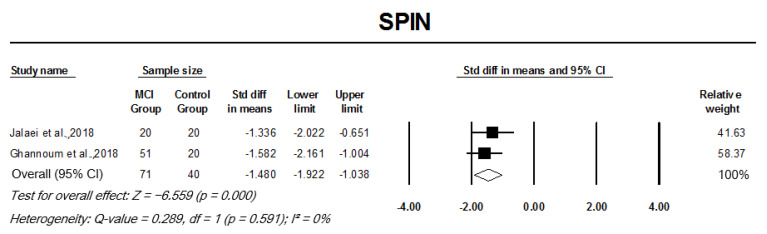
Forest plot of standard mean difference and overall (pooled) estimate of studies investigating speech perception in noise (SPIN) test that compared a mild cognitive impairment (MCI) group to a control group. Notes: Z = Z score; *I*^2^ = percentage of heterogeneity; Q-value = Cochrane’s Q; df = degrees of freedom. The horizontal lines represent the 95% confidence interval (CI) for each computed standard mean difference. Weights are from the random-effects analysis.

**Figure 7:**
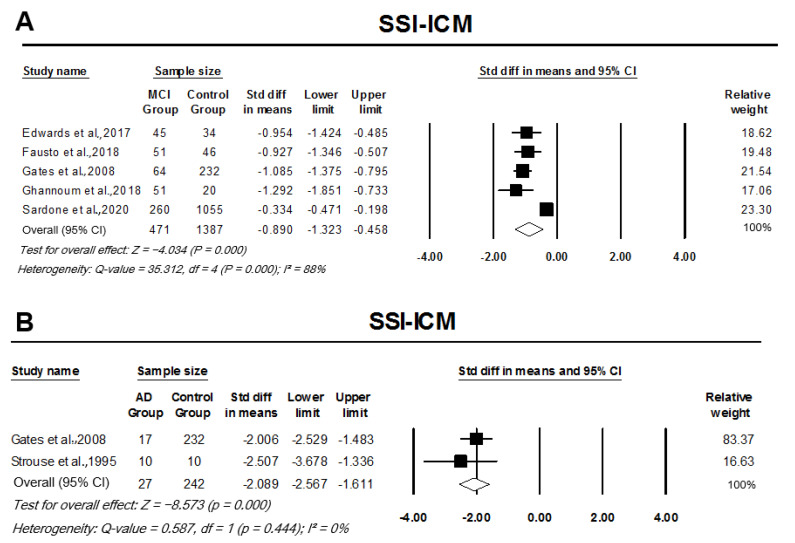
Forest plot of standard mean difference and overall (pooled) estimate of studies investigating synthetic sentence identification-ipsilateral competing message (SSI-ICM) test. (**A**) Analysis of SSI-ICM between the mild cognitive impairment (MCI) group and the control group; (**B**) analysis of SSI-ICM between the Alzheimer’s disease (AD) group and the control group. Notes: Z = Z score; *I*^2^ = percentage of heterogeneity; Q-value = Cochrane’s Q; df = degrees of freedom. The horizontal lines represent the 95% confidence interval (CI) for each computed standard mean difference. Weights are from the random-effects analysis.

**Figure 8:**
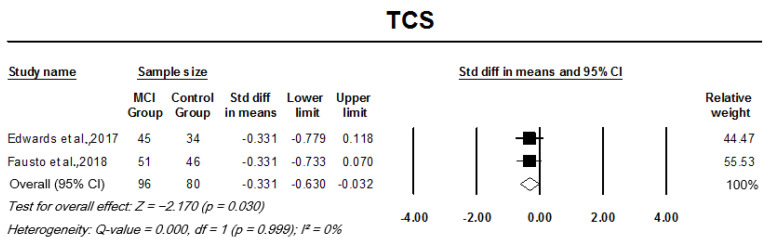
Forest plot of standard mean difference and overall (pooled) estimate of studies investigating time-compressed speech (TCS) test (average of 45% and 65% compression) that compare a mild cognitive impairment (MCI) group to a control group. Notes: Z = Z score; *I*^2^ = percentage of heterogeneity; Q-value = Cochrane’s Q; df = degrees of freedom. The horizontal lines represent the 95% confidence interval (CI) for each computed standard mean difference. Weights are from the random-effects analysis.

Pooled analysis of monaural low-redundancy speech tests indicated a significant SMD between patient groups and controls. MCI participants had significantly lower mean scores for SPIN testing when compared to controls, with the analysis indicating a large effect size, SMD −1.48 (95% CI: −1.92–−1.03, *p* = 0.000; Figure 6). MCI participants scored 4.034 standard deviations below the mean of controls in SSI-ICM, indicating a large effective size, pooled SMD, −0.89 (95% CI: −1.32–−0.46, *p* = 0.000; Figure 7A). Similarly, AD participants also performed significantly worse in SSI-ICM testing when compared to controls, pooled SMD, −2.09 (95% CI: −2.57–−1.61, *p* = 0.000; Figure 7B). Although pooled analysis of TCS studies indicated a relatively small effect size when comparing MCI participants to controls, their difference was significant (SMD −0.33, 95% CI: −0.63–0.03, *p* = 0.030). There was no heterogeneity across studies in the analysis of any of the monaural low-redundancy speech tests except for MCI studies on SSI-ICM ($I^{2}$ = 88%, *p* = 0.000) (refer to Figure 6, Figure 7 and Figure 8).

### 3.4. Quality Assessment

A total of 12 of the 13 studies analysed using the quantitative tool (EPHPP, 1998) were rated “moderate”, and one was rated “weak”. None of the 13 studies included met all the core components of the quality assessment, which can be attributed to the absence of information or lack of clarity (refer to Table 2). None of the studies were described as randomised trials or indicated that blinding was used in the study design. Overall, all studies were deemed to have used reliable and valid collection tools and appropriate statistical analysis methods for the study design (Table 2).

## 4. Discussion

This systematic review and meta-analysis aimed to review studies that examine central auditory processing function in AD and its preclinical stages using behavioural central auditory processing tests. Meta-analyses were conducted on temporal auditory processing tests (ATF and ATTR), dichotic tests (DDT and DSI), and monaural low-redundancy speech tests (SSI-ICM, TCS, and SPIN). Results from this investigation suggest that participants with MCI performed significantly worse than healthy controls within channel ATTR, DDT, DSI, SPIN, SSI-ICM, and TCS central auditory processing tests. In addition, this analysis indicates that participants with AD performed significantly worse than healthy controls in DDT, DSI, and SSI-ICM central auditory processing assessments.

### 4.1. Auditory Temporal Processing in MCI and AD

Temporal auditory processing refers to the perception and processing of sound or the changes of durational characteristics of sound within a particular time interval [54]. The ability to identify, process, and sequence auditory patterns has been suggested to involve several processes which require information integration from both hemispheres across the corpus callosum [55]. These processes also involve cognitive abilities such as attentive executive functions and memory [56,57]. Pooled analysis of the available literature illustrates the association between cognitive impairment and impairment in some aspects of temporal processing. Previously, older adults have been shown to perform worse in both subtests of ATTR (within-channel and across-channel) in comparison to younger adults, as temporal processing has been demonstrated to decline with age [58,59]. However, pooled analysis indicates that participants with MCI show impairment in the within-channel gap detection task (within channel ATTR), but not in the across-channel task. This suggests that within-channel gap detection is more impaired in people with cognitive impairment (MCI) than can be attributed to normal aging alone. Although ATF is thought to be a measure of temporal resolution, pooled analysis of the ATF test indicates that there is no significant difference in performance of this test when comparing MCI participants and healthy age-matched controls, suggesting that ATF may not be an accurate measure of temporal resolution in cognitively impaired older adults.

A limited number of studies investigated GIN, RGDT, and PPS in participants with cognitive impairment. Therefore, pooled analysis was not possible. This could be attributed to the complexity of these behavioural tests and the difficulty to perform in people with cognitive impairment. These tests require complex responses from the individuals performing the test and require substantial attention, understanding, and participation, limiting their use and reliability in people with moderate-to-severe cognitive impairment.

### 4.2. Dichotic Tests/Binaural Interaction in MCI and AD

Dichotic listening tasks are suggested to involve cognitive processes such as memory and attention. Several neuroimaging studies show frontal, temporal, and parietal lobe activation when performing these tasks [60,61,62], indicating the involvement of a cortical network of interacting cerebral regions mediating attention in dichotic listening [63]. The meta-analyses of dichotic listening tasks, DDT and DSI, indicate that participants with MCI and AD show decreased performance on these tasks compared to age-matched controls. Previous findings have found an association between dichotic performance in AD and cortical atrophy, particularly in the temporal lobe [64,65].

In addition, DSI performance has also been related to cortical thickness in the inferior gyrus and auditory transverse and superior temporal gyri, which are involved in language and auditory function [66]. In addition, several imaging studies have found that early changes in the cortical thickness of the inferior gyrus and auditory superior and transverse temporal gyri have been used as a predictor for AD [67,68,69]. DSI performance has been used to predict the reduced cortical thickness of the middle frontal gyrus, a brain region linked to episodic and working memory, which are often impaired in cognitively impaired elderly [70].

Participants with AD often have impaired executive functions, resulting in an inability to respond accurately in the dichotic listening task when instructed to focus attention on a particular ear [71,72,73]. Most of the studies included in this review used free recall methods for the dichotic listening task rather than focused-attention conditions. It is suggested that free recall conditions use bottom-up processing of verbal stimuli, which rely heavily on the structural hemispheric difference in language processing; this, in turn, results in superior performance in the right ear [63]. This is further supported by the results reported in most of the reviewed articles, with cognitively impaired individuals scoring higher in the right ear in both DDT [72,74,75,76] and DSI [36,75], indicating impaired cortical attentional networks in MCI and AD participants. Changes in frontal pathology in MCI and AD participants may lead to decreased attentional and executive functions, resulting in increased impairment to attend to stimuli presented to the left ear than to the right ear [72].

### 4.3. Monaural Low-Redundancy Speech in MCI and AD

The findings in this meta-analysis indicate that SSI-ICM performance is impaired in participants with MCI and AD. It has been previously demonstrated that the SSI-ICM score is associated with parahippocampal gyrus and entorhinal cortical thickness, regions susceptible to atrophy in pre-symptomatic stages of AD [77]. In addition, SSI-ICM performance has also been linked to cortical thickness in the inferior parietal lobe. This region is involved in sensory integration and is often impacted by neurodegeneration in individuals with AD [78]. Reduced performance on SSI-ICM in MCI and AD participants can also indicate impaired auditory processing in these individuals, as there is a strong correlation between SSI-ICM score and cortical thickness of the Heschl’s gyrus, commonly known as the primary auditory cortex [79].

Deficient speech perception in noise has been suggested to be associated with decreased neural synchrony, which in turn leads to impaired processing of timing information in noise [80]. The temporal resolution has also been identified to play an important role in perception. In addition, temporal information is crucial for object identification and subsequent sound segregation [80,81,82]. Previous research indicated that excessive noise-induced neural delays obstruct a listener’s ability to extract a particular signal from background noise, interfering with stream segregation at the brainstem and cortical levels, resulting in poorer SPIN [80]. Pooled analysis has shown that MCI participants performed significantly poorer than the control group in the ability to preserve words in the presence of noise. Anatomical pathways that play a role in distinguishing useful signals from noise lie in the medial olivary complex. The modulation of the medial olivary complex on the outer hair cells to reduce the gain of noisy signals is primarily activated in the dorsolateral prefrontal cortex area, which is involved in attentive-executive functions [83]. It has been previously demonstrated that deficits in the dorsolateral prefrontal cortex are associated with working memory, which is impaired in participants with cognitive decline [84]. Poorer performance in SPIN or TCS could reflect dorsolateral prefrontal cortex deficits in participants with MCI.

### 4.4. Auditory Memory and Discrimination in MCI and AD

Pooled analysis was not possible for auditory memory tests due to the limited number of studies that included GFW in the test battery for participants with MCI and AD. One study showed that recognition memory, auditory memory for content, and auditory memory for sequence were all significantly impaired in participants with MCI compared to healthy age-matched controls when using the GFW test battery [74]. The GFW test battery has been used to evaluate how an individual manages a particular task that requires short-term memory and the ability to redirect attention rapidly [74,85]. While it is known that attention and memory are impaired in MCI and AD participants, more research is required to conclude the utility of GFW as an accurate test of auditory memory or whether it has the potential to be used as a screening tool for cognitive impairment.

Similarly, this review was unable to assess tone duration discrimination due to the limited number of studies that used this test in participants with cognitive impairment. TDD has been previously demonstrated to be impaired in participants with AD compared to healthy controls [86]. The poorer performance of AD participants on the TDD task may indicate slowed processing of the durational properties of an acoustic signal [87]. This implies a possible disadvantage for cognitively impaired individuals in discriminating durational changes in complex and rapidly changing acoustic signals encompassing everyday conversational speech [87].

Due to aging, pathophysiological changes occur in the peripheral and central auditory systems [9]. Several genetic, environmental, and health co-morbid factors increase the risk of age-related peripheral hearing loss and further influence the changes in the central auditory pathway [9]. Even though several theories have been proposed to explain the association between peripheral hearing loss and dementia, the causal association between central auditory impairment and dementia requires further clarifications. These clarifications are essential for designing diagnostic assessments and treatment strategies. The ‘central effect of biological aging (CEBA)’ hypothesis posits that age-related changes in the central nervous system (both auditory and non-auditory regions) without accompanying peripheral deficits could result in central auditory processing impairment [88]. In this situation, individuals would exhibit normal or near-normal pure-tone audiograms with an increasing number of complaints on the difficulty in understanding speech in the presence of background noise.

The ‘central effects of peripheral pathology’ (CEPP) hypothesis argues that the pathophysiological changes in the central auditory pathway result from peripheral hearing impairment [88]. CEPP could lead to information degradation [89,90,91], increased listening effort [92] and increased cognitive load [89,93]. CEBA can occur without CEPP and vice versa, and both can co-exist simultaneously [88]. This study observed a significant relationship between central auditory impairment cognitive impairment due to underlying neurodegenerative conditions, supporting the ‘common cause’ [89,93,94] hypothesis. Humes et al. 2012 [95] proposed that multiple conditions resulting from age-or-disease-related conditions affecting the auditory pathway and the brain could manifest as CAP impairment. Hence, it is important to be mindful that CAP impairment due to underlying neurodegenerative diseases could co-exist with CEBA and/or CEPP.

Suppose the CAP impairment is due to CEPP. In that case, the first solution should be providing suitable amplification devices to remedy the peripheral hearing pathology, such as hearables, hearing aids, or hearing implants. Suppose the CAP impairment is due to CEBA with no peripheral impairment yet causing challenges in speech understanding in background noise. In that case, personal assistive listening devices such as hearing loop (or induction loop) systems, FM systems, infrared systems, amplified telephones, notification systems, personal amplifiers, TV streamers, and captions could help deal with background noise. Further auditory training to help discriminate between tones, noises, digits or speech sounds, sound localisation, or focus on other hearing-related skills could also be helpful [96].

### 4.5. Clinical Limitations of the CAP Testing

There are several limitations in conducting CAP testing with older adults in clinical settings. CAP testing cannot be performed in older adults with moderately severe-to-profound sensorineural hearing loss. Even less severe peripheral hearing loss can affect CAP testing results, mainly when testing speech discrimination in the presence of background noise. This is further complicated as binaural separation, binaural integration, and monaural tasks cannot be performed in the free field. Therefore, it is not possible for those who wear hearing aids or cochlear implants to use them while testing. Peripheral hearing loss can be mitigated by presenting tests at the client preferred level and using closed set testing such as DDT and SSI-ICM or tests such as DPT that are not dependent on speech discrimination. Future studies may conduct speech in noise testing, such as the SSI-ICM in the free field to wear hearing devices during testing.

Additionally, the results can vary from one CAP test to another on the same person. Using a CAP test battery that assesses a multitude of auditory processing skills while considering peripheral hearing and cognitive loading will reflect the most accurate assessment of an individual’s auditory processing skills in clinical settings. However, it is vital to consider the length of the test battery to ensure that it is not too long; otherwise, results may be confounded by fatigue. Other health-related factors could affect test outcomes that can influence the test results, i.e., vision, physical, mental, and psychological. As the CAP testing requires active participation, it would be difficult to obtain reliable test results from those diagnosed with dementia. Incorporating electrophysiological test measures such as P300 could be helpful when assessing CAP impairment in older adults diagnosed with dementia.

Many current studies compare mean scores of SCD, MCI, and AD groups to a healthy control group. However, to be able to develop a CAP assessment/test battery that is accurate and is a sensitive early detection tool for AD, further development needs to consider how a healthcare professional can implement the test battery on an individual when considering the fact that it is normal for individuals to fail some tasks in a CAP test battery. There has been much debate within the audiology profession about which CAP results constitute a CAP impairment diagnosis. This will be the same difficulty faced when developing a clinically implementable CAP battery as a screening tool for early indication of SCD, MCI, or AD.

The outcomes of the auditory-verbal CAP test rely not only on factors related to cognition and hearing but also on linguistic factors. Therefore, fluency and accent can also impact the outcomes of these tests. Some of the auditory–verbal CAP tests are validated only in a few languages. Consequently, they cannot be used with non-native speakers.

## 5. Conclusions

Few studies have investigated the central auditory processing functions of those diagnosed with AD. These central auditory processing tests are behavioural tests that are difficult to perform in people with AD, which could explain the limited number of studies with AD participants that look at central auditory processing using behavioural tests. Due to the limited number of studies that investigate CAP behavioural assessments in people with MCI compared to AD, this analysis was unable to examine whether any of these assessments can differentiate between participants with AD and MCI. This analysis indicated that MCI participants could be differentiated from healthy aged-matched controls on the basis of their performance on some of the behavioural CAP assessments. In conclusion, a subjective CAP test battery can be used as a hearing biomarker/clinical tool to early identify older adults at risk of cognitive impairment in clinical settings.

## Figures and Tables

**Table 1:** Data extraction summary of central auditory assessment studies included in the systematic review.

Study (Country)	Groups Mean Age (±SD) (n) M/F	Patient Diagnostic Tool	Study Aim(s)	Types of Tests Used	Outcomes Measured	Major Findings	Limitations and/or Difficulties Reported
Edwards et al., 2016 USA	MCI: 73.73 ± 6.82 (45) 30/15	MoCA	To compare older adults with and without MCI in auditory performance in competing acoustics signals and temporal aspects of audition.	SSI-ICM	Percent of correct answers	MCI < HC (*p* < 0.001)	Thorough neuropsychological evaluations for all study participants could not be obtained.
				DSI (Free recall)	Percent of correct answers	MCI < HC (*p* < 0.001)	
	HC: 70.59 ± 5.77 (34) 11/23			ATTR (Across channel and within channel)	Average of shortest gap detected by participants (ms)	Across channel: MCI = HC	The sample included community-dwelling, noninstitutionalised older adults who were required to commute to the location of testing and were likely less impaired than the population.
						Within channel: MCI > HC (*p* < 0.05)	
				TCS (Presented at a compression rate of 45% and 65%)	Percent of correct answers: average of score at 45% and 65% compression	MCI = HC	
Fausto et al., 2017 USA	MCI: 74.53 ± 6.58 (55) 30/21	MoCA	To compare the Cognitive Self-Report Questionnaire (CSRQ) Hearing and Cognitive subscale ratings among older adults with and without MCI	SSI-ICM	Percent of correct answers	MCI < HC (*p* < 0.05)	The study did not examine cognitive domains other than memory.
				DSI (Free recall)	Percent of correct answers	MCI < HC (*p* < 0.05)	
				ATTR (Across channel and within channel)	Average of shortest gap detected by participants (ms)	Across channel: MCI = HC	Only assessed speech understanding in single-talker competition and did not assess speech understanding in multi-talker or broadband noise.
	HC: 71.37 ± 6.09 (50) 18/26		To examine whether self-report, as measured by the CSRQ, is associated with objective measures of hearing, auditory processing, and cognition.			Within channel: MCI > HC (*p* < 0.05)	
				TCS (Presented at a compression rate of 45% and 65%)	Percent correct out of 100	45% compression: MCI = HC	Lack of a diverse sample population
						65% compression: MCI = HC	
Gates et al., 2008 USA	AD: 84.0 ± 5.1 (17) 10/7	CASI, CDR, and NINCDS-ADRDA	To evaluate whether abnormal central auditory processing test results could also be observed in persons with memory loss but none of the other criteria for a diagnosis of AD (i.e., MCI).	SSI-ICM	Percent of correct answers	MCI < HC (*p* < 0.05)	Patients must have sufficient vision to read the number of sentences heard and sufficient peripheral auditory function to understand speech at a comfortable loudness level. Because of the need to ensure adequate peripheral auditory function, CAP testing would not be suitable for those with severe hearing losses.
						AD < HC (*p* < 0.05)	
	MCI: 82.3 ± 6.1 (64) 23/41			DSI (Free recall)	Percent of correct answers	MCI < HC (*p* < 0.05)	
						AD < HC (*p* < 0.05)	
	HC: 78.8 ± 4.7 (232) 86/146			DDT (Free recall)	Percent of correct answers	MCI < HC (*p* < 0.05)	
						AD < HC (*p* < 0.05)	
Ghannoum et al., 2018 Egypt	MCI: 59.35 ± 4.8 (51) 34/17	DSM-V	To clarify if the cognitive decline is associated with central auditory dysfunction.	SSI-ICM	Percent of correct answers	MCI < HC (*p* < 0.001)	None reported
			To assess which tests of central auditory dysfunction or function of central auditory processing should be included in the early diagnostic procedure of memory complaints.	SSI-CCM	Percent of correct answers	MCI < HC (*p* < 0.001)	
				DDT (Free recall)	Percent of correct answers	MCI < HC (*p* < 0.002)	
	HC: 58.15 ± 4.18 (20) 13/7		To identify which type of CAP impairment is present in patients with cognitive impairment.	AFT	ms	MCI > HC (*p* < 0.001)	
			To correlate objective auditory evoked potentials in speech auditory brainstem response with cognitive and central auditory dysfunction.	GFW	Correct number of words recalled	MCI < HC (*p* < 0.001)	
				SPIN	Percent of correct answers (word recall)	MCI < HC (*p* < 0.001)	
Gootjes et al., 2018 The Netherlands	AD: 69.3 ± 8.7 (25) 18/7	NINCDS-ADRDA	The study aimed to see whether asymmetrical performance on a dichotic listening task (DLT) in Alzheimer’s disease and aging is related to white matter pathology as reflected by corpus callosum atrophy.	DDT (DLT) (Directed attention)	Number of correct responses out of 60	AD < SCD (*p* < 0.001)	Several patients had profound difficulties attending to the LE, and the attentional deficits of this subgroup might contaminate possible associations.
	SCD (SMC): 66.1 ± 9.3 (20) 13/7					AD< HC (*p* < 0.05)	
	HC: 68.6 ± 9.1 (20) 9/11					SCD = HC	
Hellstrom et al., 1996 Sweden	AD: 72.5 ± 6.5 (29) 6/23	NINCDS-ADRDA and DSM-III-R	To investigate to what extent groups of AD, Ml, and healthy elderly can be differentiated by a TDD test.	TDD	C% Total percent correct responses	AD < HC (*p* < 0.05)	None reported.
	MI (MCI): 73.6 ± 7.9 (10) 4/6					MI < HC (*p* < 0.05)	
	HC: 80.6 ± 3 (21) 9/12						
Lliadou et al., 2016 Greece	MCI: 51-82 (18) 6/12	DSM-V	To evaluate auditory perception in a group of older adults diagnosed with mild cognitive impairment (MCI).	SinB	SNR of 50% correct speech identification	MCI < HC (*p* < 0.05)	None reported.
				RGDT	Threshold of gap detection at each frequency (shortest time interval participants reports perception of two tones)	MCI < HC (*p* < 0.005)	
	HC: 50-73 (11) 5/6			GIN	Gap detection threshold (shortest gap duration detected on at least four out of six presentations)	MCI < HC (*p* < 0.01)	
Jayakody et al., 2020 Australia	SCD (SMC) 71.5 ± 7.2 (61) 20/41 HC 68.8 ± 7.7 (34) 10/24	MAC-Q and MoCA	To examine the central auditory processing (CAP) assessment results of adults between 45 and 85 years of age with subjective memory complaints (SMCs) as compared to those who were not reporting significant levels of memory complaints (non-SMCs).	DDT (Free recall)	Percent of correct answers	SCD = HC	None reported.
				DPT	Percent of correct answers	SCD = HC	
				QuickSIN	Signal-to-noise ratio loss	SCD = HC	
				DSI (Directed attention)	Percent of correct answers	SCD = HC	
				SSI-ICM	Percent of correct answers	SCD < HC (*p* < 0.05)	
Jalaei et al., 2019 Iran	MCI: 70.75 ± 5.09 (20) 13/7	MMSE score	The purpose of this study was to examine the utility of central auditory processing tests as early diagnostic tools for identifying the elderly with MCI.	SPIN	Percent of correct answers (word recall)	MCI < HC (*p* < 0.001)	The use of simple clinical measures to investigate sensory processing is not enough to detect the sensory impairment associated with cognitive impairment. Moreover, frequency discrimination and temporal processing are needed for better speech perception.
	HC: 71.3 ± 4.41 (20) 12/8			GIN	Gap detection threshold (the smallest gap that the subject detects correctly in at least four out of the six presentations)	MCI > HC (*p* < 0.001)	
Lee et al., 2018 Korea	MCI: 68.56 ± 6.34 (30) 6/24	Petersen’s criteria and MMSE score	The purpose of this study was (1) to compare speech perception performance among MCI subgroups and (2) to identify the cognitive domains specifically related to speech-in-noise perception.	SPIN	Percent of correct answers (word recall)	MCI < HC (*p* < 0.05)	None reported.
	HC: 63.92 ± 4.48 (39) 14/25						
Rahman et al., 2011 Egypt	MCI: 66.5 ± 5.4 (150) 70/80	CAMCOG	To assess if central auditory processing skills are affected in patients with MCI or not and assess sensitivity and specificity of central auditory processing tests in the detection of MCI.	SAAT	Percent of correct answers	MCI < HC (*p* = 0.001)	CAP tests require patients to be attentive and have sufficient peripheral auditory function to understand speech at a comfortable loudness level.
				DDT (Free recall)	Percent of correct answers	MCI < HC Left ear only (*p* = 0.005)	
	HC: 66.4 ± 5.6 (150) 70/80			AFT	ms	MCI = HC	
				PPS	Percent of correct answers	MCI < HC (*p* = 0.002)	
				GFW	Correct number of words recalled	MCI < HC (*p* = 0.001)	
Sardone et al., 2020 Italy	MCI: 74 ± 5.62 (260) 148/112	DSM-V	To explore the associations of age-related central auditory processing disorder (CAPD) with mild cognitive impairment (MCI) and dementia in an older population-based cohort.	SSI-ICM	Percent of correct answers	MCI < HC (*p* < 0.05)	None reported.
	HC: 73.1 ± 5.74 (1055) 535/520						
Strouse et al., 1995 USA	AD: 72.3 ± 11.6 (10) 2/8	DSM-III-R	To determine whether people in the early to middle phases of AD show impaired central auditory processing than those without dementia.	SSI-ICM	Percent of correct answers	AD < HC @ 0dB, −10 dB, −20 dB (*p* = 0.0001)	One subject within each experimental group was below the age of 65, and thus comparisons with existing studies evaluating elderly populations would not be applicable for these subjects.
				DSI (Free recall)	Percent of correct answers	AD < HC (*p* < 0.004)	
	HC: 70.1 ± 7.9 (10) 2/8			DDT (Free recall)	Percent of correct answers	AD < HC (*p* < 0.001)	
				PPS	Percent of correct answers	AD = HC	
				DPT	Percent of correct answers	AD < HC (*p* = 0.0001)	

Abbreviations: AD = Alzheimer’s group, MCI = mild cognitive impairment group, SCD = subjective cognitive decline group, HC = healthy controls (aged matched), CASI = cognitive ability screening instrument, CDR = clinical dementia rating, NINCDS-ADRDA = National Institute of Neurological and Communicative Diseases and Stroke–Alzheimer Disease and Related Disorders Association criteria, DSM = Diagnostic and Statistical Manual of Mental Disorders, MMSE = Mini-Mental State Examination, MoCA = Montreal Cognitive Assessment, MAC-Q = Memory Assessment Clinics Questionnaire, AFT = Auditory Fusion Test, SSI-ICM = synthetic sentence identification-ipsilateral competing message, ATTR = adaptive tests of temporal response, DDT = Dichotic Digits Test, DSI = dichotic sentence identification, DLT = dichotic listening task, TCS = time compressed speech test, FS = filtered speech test, TDD = tone duration discrimination, SinB = speech in Babble, GIN = gap-in-noise, RGDT = Random Gap Detection Test, SNR = signal to noise ratio, SPIN = speech perception in noise, CAMCOG = Cambridge Cognitive Examination, SAAT = selective auditory attention test, PPS = pitch pattern sequence, GFW = auditory memory battery of Goldman–Fristoe–Woodcock, DPT = duration pattern test, QuickSIN = Quick Speech-in-Noise.

**Table 2:** Qualitative assessment results for quantitative studies included in the review (*n* = 13).

Core Item	Tool Question (EPHPP, 1998)	Studies with Positive Assessment
**Selection Bias**	Are the individuals selected to participate in the study likely to be representative of the target population?	11 (very likely)
	What percentage of selected individuals agreed to participate?	2 (80–100%) *11 (not described)*
**Study Design**	Was the study described as randomised? If NO, go to CONFOUNDERS. If Yes, was the method of randomisation described? If Yes, was the method appropriate?	0 (Yes)
**Confounders**	Were there important differences between groups before the intervention?	9 (No)
	Indicate the percentage of relevant confounders that were controlled either in the design (e.g., stratification, matching) or analysis.	13 (80–100%)
**Blinding**	Was (were) the outcome assessor(s) aware of the intervention or exposure status of participants?	0 (No)
	Were the study participants aware of the research question?	0 (No)
**Data Collection Methods**	Were data collection tools shown to be valid?	13 (Yes)
	Were data collection tools shown to be reliable?	13 (Yes)
**Withdraws and Dropout**	Were withdrawals and dropouts reported in terms of numbers and/or reasons per group?	Not applicable *(all studies were retrospective case–control)*
	Indicate the percentage of participants completing the study. (If the percentage differs by group, record the lowest.)	Not applicable *(all studies were retrospective case–control)*
**Intervention Integrity**	What percentage of participants received the allocated intervention or exposure of interest?	13 (80–100%)
	Was the consistency of the intervention measured?	13 (Yes)
	Is it likely that subjects received an unintended intervention (contamination or cointervention) that may influence the results?	13 (No)
**Analysis**	Indicate the unit of allocation.	13 (Individual)
	Indicate the unit of analysis.	13 (Individual)
	Are the statistical methods appropriate for the study design?	13 (Yes)
	Was the analysis performed by intervention allocation status (i.e., intention to treat) rather than the actual intervention received?	13 (No)

## Data Availability

The data presented in this study are available on request from the corresponding author.
